# Evaluating Robotic Walker Performance: Stability, Responsiveness, and Accuracy in User Movement Detection

**DOI:** 10.3390/s25113428

**Published:** 2025-05-29

**Authors:** Larisa Dunai, Isabel Seguí Verdú, Sui Liang, Ismael Lengua Lengua

**Affiliations:** 1 Department Graphical Engineering, Universitat Politècnica de València, 46022 Valencia, Spain; issever@doctor.upv.es (I.S.V.); sliang1@doctor.upv.es (S.L.); 2 Centro de Investigación en Tecnología Gráfica, Universitat Politècnica de València, 46022 Valencia, Spain; ilengua@upv.es

**Keywords:** robotic walker, gait analysis, assistive technology, sensors, inertial measurement unit (IMU), time-of-flight (TOF) sensors, principal component analysis (PCA)

## Abstract

This work presents the experimental evaluation of a robotic walker following the full implementation of its sensor and motorization system. The aging population and increasing mobility impairments drive the need for assistive robotic technologies that enhance safe and independent movement. The main objective was to validate the device’s behavior in real-use scenarios by assessing its stability, responsiveness, and accuracy in detecting user movement. Tests were carried out in straight-line walking and on paths involving directional changes, both with and without motor assistance, using a cohort of five test users. Principal Component Analysis (PCA) and t-SNE dimensionality reduction techniques were applied to analyze the inertial (IMU) and proximity (TOF) sensor data, complemented by motor control monitoring through wheel Hall sensors, to explore gait patterns and system performance. Additionally, synchronized measurements between the user’s and walker’s inertial units and Time-of-Flight sensors allowed the evaluation of spatial alignment and motion correlation. The results provide a foundation for future system adjustment and optimization, ensuring the walker offers effective, safe, and adaptive assistance tailored to the user’s needs. Findings reveal that the walker successfully distinguishes individual gait patterns and adapts its behavior accordingly, demonstrating its potential for personalized mobility support.

## 1. Introduction

The aging of the global population and the increasing prevalence of mobility impairments have driven the development of intelligent assistive technologies to support independent movement. In this context, robotic walkers have emerged as promising solutions, integrating advanced sensing, control, and user-interaction systems to enhance safety and mobility. These devices represent a significant evolution in assistive technologies, combining artificial intelligence (AI), real-time sensor data processing, and smart actuation to adapt to the user’s gait and environmental conditions [1,2].

### 1.1. Background and Related Work

Recent years have seen significant advancements in robotic walker design, combining artificial intelligence, real-time sensor fusion, and smart actuation [3]. Modern robotic walkers are equipped with multi-modal sensor systems, including proximity sensors, sonar, infrared, Time-of-Flight (TOF), laser range finders (LRF), inertial measurement units (IMU) [4], and cameras, that enable improved environmental perception, user tracking, and interaction monitoring [5,6]. Devices such as XR4000 [7], GUIDO [8], ASBGo++ [9,10], and MOBOT [11] have demonstrated the utility of sonar and infrared sensors for obstacle detection and indoor navigation. At the same time, LRF-based systems like CAIROW [12] and COOL Aide [13] offer enhanced directional estimation and route planning. As integrated in ISR AI Walker [12] and PAMM [14,15], cameras provide real-time trajectory tracking and user state recognition, improving navigational precision and safety [4].

Additionally, integrating force sensors into handles and structural supports has enabled robotic walkers to assess user stability and provide adaptive assistance during critical tasks, such as sit-to-stand transitions [16]. For instance, systems like i-Walker [17] and Simbiosis Walker [18] measure interaction forces to detect postural intent and adjust motor outputs in real time, enhancing safety and reducing fall risk. Vision-based input devices, such as the Leap Motion Controller, have also been introduced to allow intuitive gesture-based interaction, offering a non-invasive interface that is handy for users with limited upper-limb mobility [19].

While this study focuses on non-invasive sensing technologies such as IMU and TOF sensors, it is important to acknowledge that other modalities, particularly surface electromyography (EMG), have also been explored in recent literature for gait phase detection and prosthetic control [20,21]. These approaches provide valuable insights, especially for applications requiring neuromuscular-level signal decoding. However, EMG-based systems typically require controlled conditions, skin preparation, and expert placement, which makes them less suitable for real-world assistive walker scenarios involving older adults or non-clinical environments. For these reasons, EMG was not included in our system design. Nonetheless, to reflect an awareness of current research directions, we briefly reference one recent work on EMG and kinematic signal fusion.

Although robotic walkers have achieved significant progress, challenges remain regarding their influence on gait dynamics during assisted movement. Wan and Yamada [1] showed that the intervention of a robotic walker altered the determinism of user gait, particularly along the anterior–posterior direction, suggesting an impact on the natural stability and predictability of movement. Furthermore, recent developments in innovative walker control systems [22] highlight that while walkers can track user trajectories, maintaining precise alignment in complex paths remains difficult. These observations indicate the need for further improvements to enhance the responsiveness and adaptability of robotic walkers to the dynamic characteristics of human gait.

Despite these technological improvements, robotic walkers require further advancements before achieving large-scale deployment. Key limitations include incomplete support for complex tasks (e.g., curb climbing, multi-directional movement), limited autonomy in dynamic environments, and mechanical configurations that do not fully accommodate natural human movement, particularly during sit-to-stand transitions. Most handles, for instance, are designed primarily for walking, making them suboptimal for lifting the body while standing. Studies have proposed automated systems that detect trunk inclination and user intent to assist in this transition, often relying on sagittal plane motion analysis [23].

Moreover, braking systems remain a critical aspect of safe walker operation. While many devices still rely on manual brakes like conventional rollators, others incorporate an automated brake based on gait phase detection or user proximity. This trend reflects the broader goal of developing context-aware, semi-autonomous mobility aids that adjust in real time to both environmental and user-generated cues [24].

### 1.2. Research Motivation and Objectives

In summary, robotic walkers represent a multidisciplinary innovation that combines biomechanics, robotics, sensor fusion, and human-centered design. Integrating adaptive control systems and sensor technologies enables real-time monitoring and response, offering the potential for safer and more effective gait assistance. This paper presents the experimental evaluation of an advanced robotic walker prototype, highlighting its integrated sensory and control systems, and discussing performance outcomes and areas for future improvement.

Despite recent technological advances, many robotic walkers still face challenges in accurately detecting and adapting to real-time changes in user gait and stability. These limitations can result in non-natural interaction, where the device may impose movement rather than follow or support the user’s intended pace, potentially compromising comfort and safety. A key requirement for effective assistance is the ability to synchronize with the user’s motion without pulling or pushing them during locomotion. In this work, we present an experimental evaluation of a sensor-integrated robotic walker designed to monitor user behavior through inertial and proximity sensors, aiming to improve responsiveness and movement coordination. Rather than proposing a fully autonomous solution, the study focuses on validating the technical integration and data acquisition capabilities of the system in controlled real-world scenarios.

## 2. Materials and Methods

**Figure 1:**
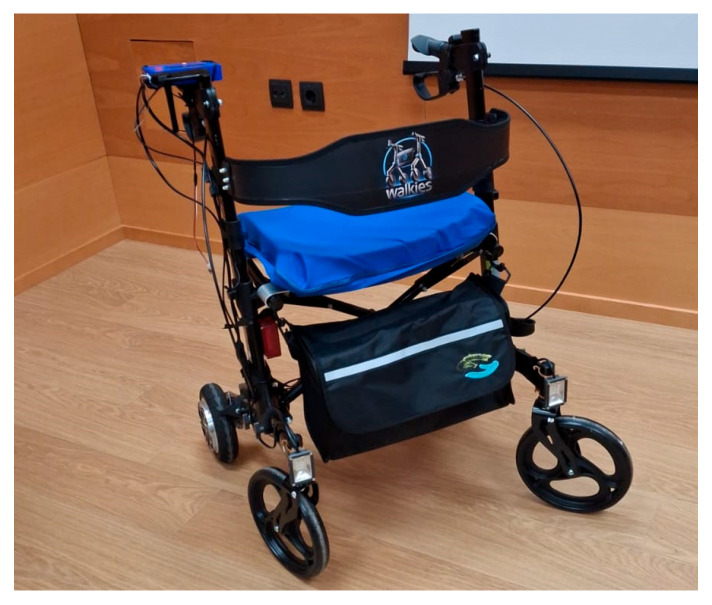
Overview of the robotic walker prototype used in the experimental evaluation, showing the integration of sensor systems (TOF sensors, IMU, and pressure sensors).

To enhance the automation, safety, and monitoring capabilities of the robotic walker and the user, the system integrates multiple sensors that enable the continuous recording and analysis of human–device interaction, as can be seen in Figure 1. The walker was equipped with strategically positioned pressure, inertial (IMU), and 4 Time-of-Flight (TOF) sensors, aimed at capturing critical data on user motion, gait behavior, and environmental interaction.

The experimental trials involved five adult participants performing controlled walking tasks under different conditions.

**Figure 2:**
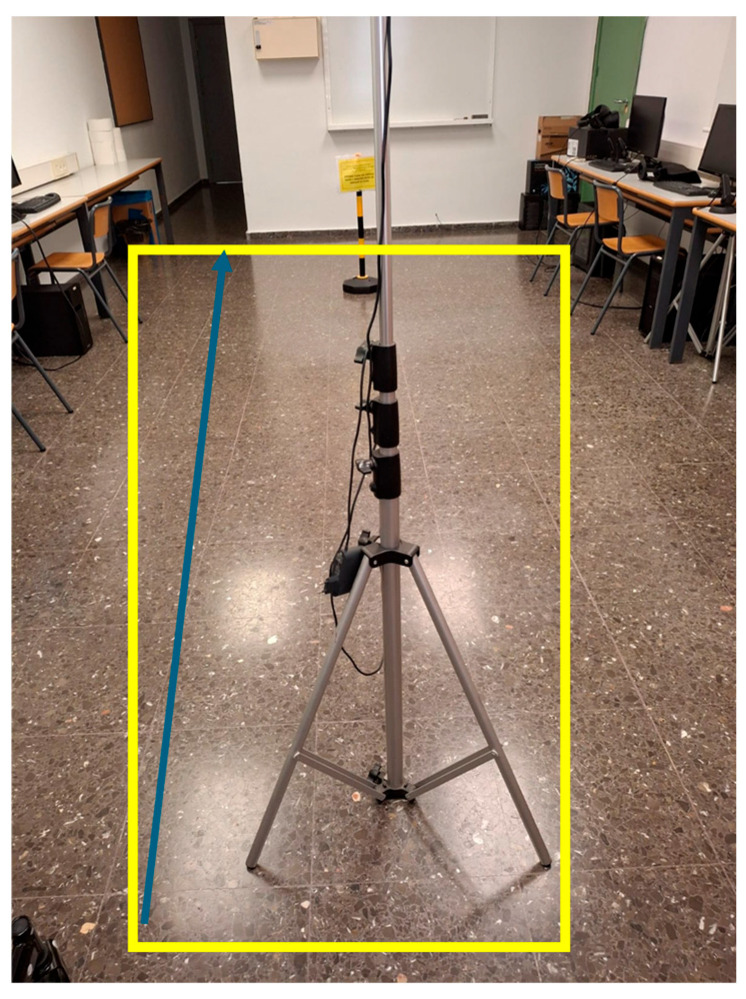
Experimental walking paths used during the trials. The yellow path represents the rectangular route (12 tiles × 4 tiles, each 40 cm), corresponding to dimensions of 4.80 m × 1.60 m. The blue path represents the straight-line route composed of 12 tiles (totaling 4.80 m). These configurations simulate typical indoor environments for assessing user–walker interaction under linear and turning conditions.

Each participant completed five trials walking in a straight line and five trials following a square path, both with motorized assistance and without it, resulting in 20 trials per participant. The straight-line path measured 2.8 m in length (7 tiles of 40 cm). The rectangular path for the square-pattern tests was constructed using 40 cm tiles, with 7 tiles forming the longer sides (2.8 m) and 5 tiles forming the shorter sides (2 m), resulting in a closed-loop layout with four 90-degree turns. This configuration was designed to simulate constrained indoor environments and evaluate user–walker interaction during direction changes (Figure 2).

This protocol ensured a balanced dataset capturing user behavior under varying movement scenarios, with consistent instructions provided for each test type.

**Figure 3:**
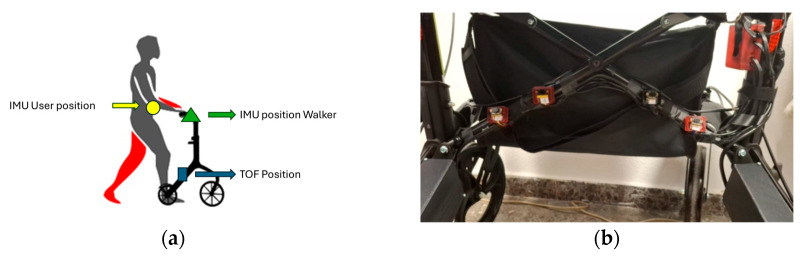
(**a**) Sensory placement on the robotic walker, showing the position and orientation of the IMU sensor and the mounting locations of the Time-of-Flight (TOF) and pressure sensors for user motion detection and environment interaction monitoring. The figure also includes the user-mounted IMU used to capture gait dynamics and body motion. (**b**) Detailed view of the real positioning of the four TOF sensors on the walker frame, illustrating their configuration for obstacle detection and distance measurement.

The complete integration and layout of the sensors were validated during the final system assembly. An overview of the sensory placement, including both the walker-mounted and user-mounted IMUs, as well as detailed positioning of the TOF sensors, is shown in Figure 3.

The selection of sensors was based on their ability to capture essential aspects of user–walker interaction. The IMU sensor was chosen to monitor orientation and linear acceleration, providing information about user posture and gait dynamics. The TOF sensors were selected for their high precision in short-range distance measurement, enabling accurate detection of obstacles and user proximity. Pressure sensors were integrated into the handles to assess user grip force and infer stability during assisted walking.

**Figure 4:**
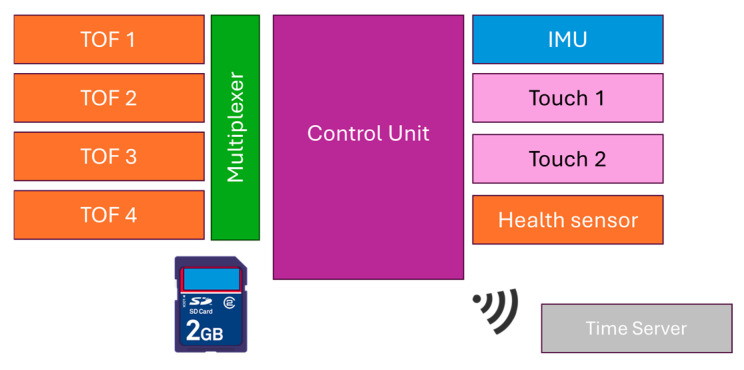
High-level schematic of robotic walker’s control architecture.

Figure 4 presents a high-level schematic of the robotic walker’s control system, highlighting the integration of all primary sensing and control modules. The system includes inertial measurement units (IMUs), Time-of-Flight (TOF) sensors, touch sensors, and health monitoring sensors, all of which are connected to a central control unit via wired interfaces. This wired configuration was deliberately selected to ensure reliable data transmission, reduce communication latency, and preserve the integrity of real-time measurements critical for gait assistance and monitoring.

To provide synchronized timestamping and accurate temporal coordination during experimental trials, a dedicated time server module is included as an independent wireless component. Its integration is designed to operate in parallel with the core sensing infrastructure, avoiding interference with the primary control and data acquisition pathways.

The architecture prioritizes real-time performance and robustness. Wired sensor interfaces were chosen to avoid the potential issues associated with wireless communication, such as transmission delays, signal degradation, or packet loss, which could compromise system responsiveness and data fidelity. Additionally, a multiplexer was employed to manage multiple TOF sensors efficiently. This allows for the sequential polling of each sensor through a single communication channel, thereby reducing hardware complexity and improving scalability without sacrificing data resolution or frequency. The Bosch TOF sensors used in this study include an internal Kalman filter as part of their signal processing firmware, according to the manufacturer’s specifications. This built-in filtering enhances the accuracy and stability of the distance measurements without requiring additional external filtering.

A display interface is included in the system for real-time data visualization and user interaction. This interface also allows for the integration of a microSD card, enabling local storage of sensor data during trials.

This modular and scalable control architecture supports dynamic and responsive operation, enabling precise monitoring and assistance under real-world conditions. Once the sensory system was assembled and validated, subsequent efforts focused on developing preprocessing routines and motion analysis techniques, as described in the following sections.

Once the robotic walker’s sensory architecture was fully assembled and validated, sensor data preprocessing and motion analysis methods were implemented, as described in the following subsections.

### 2.1. Sensor Data Preprocessing and Noise Filtering

Sensor data acquired during gait analysis are often subject to noise and environmental interference, distorting the accurate interpretation of user motion. To address this, low-pass filters were applied to the inertial data obtained from the BNO055 IMU sensor. According to the device datasheet, the embedded filters exhibit the following frequency characteristics:

- Accelerometer: embedded low-pass filter with cutoff frequency below 8 Hz (sampling at 1 kHz).
- Gyroscope: embedded low-pass filter with cutoff frequency below 12 Hz (sampling at 523 Hz).

The BNO055 is a 9-DOF (degrees of freedom) Inertial Measurement Unit (IMU) that integrates a sensor fusion algorithm to combine data from the accelerometer, gyroscope, and magnetometer, providing orientation information in quaternions and Euler angles. Its embedded low-pass filters allow low-frequency movement signals to pass while attenuating high-frequency noise.

### 2.2. Wrapping Effect Correction

One common issue in angle measurements is the wrapping effect, which occurs when angular values exceed the −180° to 180° range, resulting in discontinuities. This was addressed using the following correction formula:

(1) $$\theta corr=\theta -360{}^{\circ}=\left|\frac{\theta +360{}^{\circ}}{360{}^{\circ}}\right|$$

where *θcorr* is the corrected angle and *θ* is the measured angle.

This correction ensures continuous orientation tracking during rotational movements.

### 2.3. Kalman Filtering for Motion Prediction

Although the BNO055 includes an internal fusion filter for orientation, a Kalman filter was implemented to improve motion trajectory prediction and smooth linear acceleration data. The standard Kalman filter equations are

Prediction:

(2) $$x_{\frac{k}{k}-1}={Ax}_{k-\frac{1}{k}-1}+{Bu}_{k}$$

Correction:

(3) $$x_{\frac{k}{k}-1}=x_{\frac{k}{k}-1}+K_{k}(z_{k}-{Hx}_{\frac{k}{k}-1})$$

where *x_k/k_* state estimate at time *k*, *A* is the state transition matrix, *B* is the control input matrix, *u_k_* is the control vector, *K_k_* represents the Kalman gain and *z_k_* is the measurement vector.

This approach helps minimize measurement uncertainty and improves the robustness of user motion modeling.

### 2.4. Orientation and Acceleration Calculation

The BNO055 sensor provides quaternion and Euler angle outputs (yaw, pitch, and roll). Internally, orientation is calculated using quaternions, which can be converted to Euler angles via the following equations:

(4) $$\emptyset =arctan\frac{2{(q}_{0}q_{1}+{(q}_{2}q_{3})}{1-2\left(q_{1}^{2}+q_{2}^{2}\right)}$$

(5) $$\theta =arcsin2(q_{0}q_{2}+{(q}_{3}q_{1})$$

(6) $$\varphi =arctan\frac{2{(q}_{0}q_{3}+{(q}_{1}q_{2})}{1-2\left(q_{2}^{2}+q_{3}^{2}\right)}$$

where *q*_1_, *q*_2_, *q*_3_ are quaternion components, *Ø* is the roll angle (lateral rotation), *θ* is the pitch (front and back inclination and *φ* is the yaw rotation over the vertical axis.

Additionally, linear acceleration (*α*) is computed using:

(7) $$\alpha =\sqrt{{LiAx}^{2}+{LiAy}^{2}+{LiAz}^{2}}$$

These values are obtained directly from BNO055’s filtered output and used to assess the user’s initiation and cessation of motion.

### 2.5. Gait Event Detection and Step Length Estimation

Detecting gait events, such as heel strike and toe-off, is crucial for analyzing user mobility and extracting spatiotemporal gait parameters. In smart walkers, onboard sensors allow transparent gait monitoring without constraining the user to controlled environments or requiring wearable sensor setups [25].

During experimental trials with users, the number of steps performed was estimated by identifying heel strike events using characteristic peaks in the vertical acceleration signal (LiAz) from the user-mounted IMU. The total distance walked was recorded through the walker’s odometry system, which tracks wheel displacement via internal Hall effect sensors, and further cross-validated using Time-of-Flight (TOF) sensor measurements that capture changes in user–walker distance. These combined sensor inputs enabled the computation of related gait parameters, including step length and cadence, without requiring external motion capture systems.

As previously described, a microSD card integrated into the system’s display interface enabled the local storage of this sensor data during the trials, ensuring reliable data logging for subsequent analysis and validation.

To detect key gait events during walking, inertial sensor data were analyzed, focusing primarily on the vertical component of linear acceleration (LiAz) provided by the user-mounted IMU.

Characteristic peaks in LiAz were used to identify critical moments of the gait cycle [5]. Heel strike events were detected by identifying local maxima in the vertical acceleration signal (LiAz) from the user-mounted IMU. This method relies on the characteristic impact peak that occurs when the foot contacts the ground. A simple peak detection algorithm was applied to extract these events without further filtering, given the clear periodicity observed in the data. No supervised learning or advanced sensor fusion techniques were used for this stage of the analysis.

- Heel strike: the instant when the heel contacts the ground, marking the beginning of the stance phase. It is fundamental for detecting initial support and assessing walking stability.
- Toe-off: the instant when the toes leave the ground, initiating the swing phase. It is critical for evaluating propulsion and gait fluidity.

Detecting these events enables estimating step time, stride time, and cadence, which are standard clinical parameters to assess gait quality and rehabilitation progress [25].

Step events were segmented based on the timestamps of consecutive heel strikes. Step length (SL) was computed according to the following formula:

(8) $$Step\;Length=\frac{Total\;Distance\;Walked}{Number\;of\;Steps}$$

where the total distance walked was obtained from the displacement recorded by the walker’s odometry and verified by Time-of-Flight (TOF) sensor readings, which measured variations in the user–walker distance during locomotion.

In the developed system, the combination of Time-of-Flight (TOF) sensors and inertial data provides distance variation information relative to the walker, allowing an indirect estimation of user step initiation and cessation. Unlike traditional wearable-based systems, this approach ensures gait analysis is non-intrusive and compatible with real-world usage scenarios, similar to strategies reported in Walk-IT [25].

While IMU data were critical for accurately detecting step events and cadence estimation, TOF sensors provided complementary information about overall movement patterns and user–walker spatial dynamics, rather than individual gait events.

Deviations in step length between steps or across trials can indicate limitations in mobility, instability, or improper use of the walker. Short step lengths may reflect restricted gait patterns, whereas excessive step lengths could suggest loss of balance or excessive propulsion assistance.

**Figure 5:**
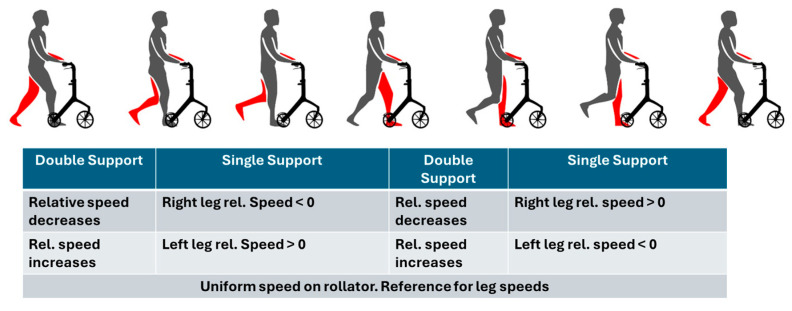
Schematic representation of the user support phases during the gait cycle relative to the walker, adapted from [26].

A schematic representation of the user support phases during the gait cycle relative to the walker is shown in Figure 5 [26].

For example, a total distance of 4.20 m with 10 steps results in a step length of 0.42 m.

Deviations in step length can indicate limitations in mobility or improper walker configuration. Too short a step length may reflect restricted gait, while excessive step length may suggest instability or misuse of the device.

In addition to basic gait event detection, more detailed analysis of the user’s walking behavior was performed by fusing sensor data from both the walker and the user, as described in the following section.

### 2.6. Sensor Fusion and Gait Behavior Analysis

To support real-time gait analysis, the robotic walker incorporates a dual-IMU configuration, with one unit mounted on the walker and the other on the user’s body. This setup enables the simultaneous and independent capture of key locomotion parameters, including

- Orientation angles (yaw, pitch, and roll);
- Linear accelerations (LIAx, LIAy, LIAz);
- Relative distance between the user and the walker.

Each IMU is independently mounted and connected to the central control system, enabling direct comparison between the user’s biomechanical motion and the walker’s mechanical adaptation. Both IMUs operate under a unified time framework, facilitated by an external wireless time server module. This module provides precise time synchronization across all sensor nodes, ensuring consistent temporal alignment for data fusion and gait parameter estimation.

The walker-mounted IMU tracks device orientation and movement compensation, while the user-mounted IMU monitors postural dynamics and stability throughout the gait cycle. Together, these measurements enable the accurate estimation of gait speed, rhythm, and balance—critical factors in delivering responsive, behavior-aware assistance.

Complementing the IMU data, Time-of-Flight (TOF) sensors continuously measure the relative position and distance between the user and the walker frame. This dual-sensor approach offers a multimodal representation of gait dynamics: IMUs provide detailed angular displacement and acceleration profiles, while TOF sensors supply spatial tracking of the user’s trajectory relative to the support structure.

Such multimodal sensing is instrumental in detecting deviations in stability, changes in user intent, and transitions between locomotor states. The system’s architecture thus supports a more adaptive and responsive assistance strategy.

Aligned with current research on wearable gait analysis systems [4], the sensor fusion approach facilitates the indirect, continuous estimation of clinically relevant metrics such as step time variability, gait speed, and double support time—without the need for laboratory-grade equipment. Moreover, recent advances in gait stability prediction, such as the step viability paradigm [3], highlight the importance of evaluating step-to-step transitions and recoverability after disturbances. While predictive algorithms were not implemented in the present prototype, the hardware configuration and synchronized data architecture are designed to accommodate future integration of such functionality.

In this way, the robotic walker functions not only as a mobility aid but also as a smart sensing platform capable of monitoring essential mobility indicators in real-world conditions. It provides a scalable foundation for future developments in adaptive assistance and personalized mobility support.

To ensure coherent and user-driven operation of all hardware components, the system includes a dedicated control logic for sequential module activation, described in the following section.

### 2.7. System Control Logic and Sensory–Actuator Interaction

The high-level control architecture of the robotic walker is designed to ensure safe, reliable, and user-driven operation by structuring the system’s activation through a sequential, hierarchical process. The system integrates multiple sensing modalities and actuator control to deliver context-aware assistance while preserving user autonomy.

Upon powering the device, an initial status check is performed using a basic ON/OFF logic gate. This first stage ensures that no subsystems are inadvertently active and that the walker remains in a safe standby state until explicitly engaged by the user.

Once activated, the system initializes the touch sensors, which serve as the primary interface for detecting user presence and intentional interaction. The control logic mandates that the user must be in contact with the walker before proceeding to activate the remaining modules. This conditional dependency on user engagement is a key safety mechanism, ensuring that the system remains inert unless human interaction is verified.

Following successful touch sensor initialization, the system sequentially activates the remaining sensor and actuator modules:

- Time-of-Flight (TOF) sensors are initialized to monitor the spatial environment, enabling obstacle detection and the estimation of user position relative to the device.
- Inertial Measurement Units (IMUs) are brought online to provide real-time data on the user’s motion, including acceleration, orientation, and gait stability.
- Motor controllers are then initialized but remain inactive until all preceding sensor modules report proper functionality.

Once the system confirms that all subsystems are operational, it transitions into motorized support mode. In this mode, sensor data from the TOF units and IMUs are continuously processed to determine the appropriate level of motor assistance. The actuator outputs are dynamically adapted to the user’s movement, enabling smooth and context-sensitive support in real time.

Crucially, continuous monitoring via the touch sensors persists throughout operation. If contact is lost or the user manually disengages, the system immediately interrupts motor output, thereby placing control fully in the hands of the user. This design ensures that the walker never operates autonomously without active user participation.

**Figure 6:**
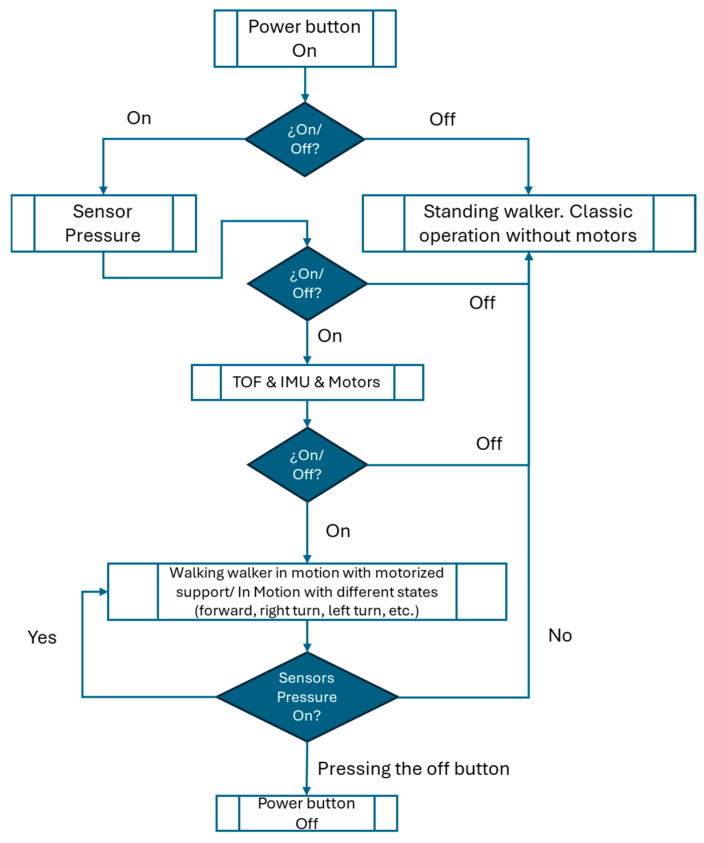
High-level flowchart of the robotic walker’s control system, illustrating the sequential activation of touch sensors, TOF sensors, IMUs, and motorized support logic.

Figure 6 presents the high-level flowchart summarizing this control logic, including the sequential activation and interdependency of sensory modules and motorized components.

- Upon switching the device, an initial status check is performed through a simple ON/OFF condition.
- If activated, the touch sensors are initialized to detect user interaction.
- Subsequent activation steps include initializing the Time-of-Flight (TOF) sensors, inertial measurement units (IMU), and motor controllers.
- If all modules are active, the walker transitions into motorized support mode, adapting its assistance according to the sensors’ input.
- The continuous monitoring of the user interaction through the touch sensor enables manual deactivation at any point, ensuring user control at all stages.

This hierarchical control structure enhances the walker’s operational safety, ensuring that motorized assistance is only provided when the user explicitly demands it and under continuous sensor supervision.

## 3. Results

Following the implementation and integration of the sensing and control systems described above, a series of experimental trials were conducted to validate the robotic walker’s performance in real-world scenarios. The collected data allowed the evaluation of gait stability, sensor responsiveness, and system adaptability during both motorized and non-motorized operation modes.

Five adult participants (three males and two females) voluntarily participated in the experimental trials. The system included two IMUs: one mounted on the walker to capture device orientation, and another worn by the user to monitor gait motion. The user-mounted IMU was placed at waist level and aligned with the sagittal plane using an adjustable strap. This placement was kept consistent across participants to ensure the comparability of the motion data. The participants ranged from 60 to 85 years, with an average height of 1.62 ± 0.06 m and an average weight of 68 ± 7 kg. All participants were autonomous walkers, presenting no severe neurological or musculoskeletal disorders, although mild gait instability was reported in one case.

Table 1 summarizes the demographic characteristics of the five participants. While the sample size is limited, this study serves as proof of concept for the technical validation of the robotic walker. Future work will involve a larger and more diverse cohort to enhance statistical significance and generalizability.

The experiments were conducted indoors under normal ambient conditions, and no additional assistive devices were used apart from the robotic walker. Written informed consent was obtained from all participants before the study.

The robotic walker was fully connected to all previously described subsystems to conduct the experimental trials. These include the motorized wheels, their respective controllers equipped with internal magnetic Hall sensors, and the TOF (Time-of-Flight) and IMU (inertial measurement unit) sensors. This integration ensured the full functionality of the robotic system during testing.

The goal of the trials was to validate the integration of the TOF and IMU sensors, assess the reliability of motor control, and verify synchronization across all collected sensor data. The outcomes from this validation process were used to optimize system performance, ensuring safe and practical assistance for the user.

**Figure 7:**
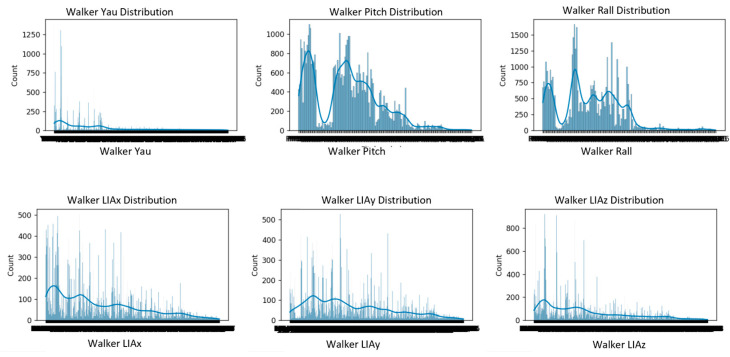
Evolution of yaw, pitch, and roll of the walker, along with LIAx, LIAy, and LIAz for the five test users.

Before applying any dimensionality reduction or correlation analysis, the evolution of the main sensor outputs (yaw, pitch, roll, and linear accelerations LIAx, LIAy, LIAz) was examined. Figure 7 shows the five test users’ temporal evolution of the orientation (yaw, pitch, roll) and linear acceleration (LIAx, LIAy, LIAz) parameters, illustrating natural variability, gait cycles, and individual interaction patterns with the walker.

Yaw signals exhibit greater variability, particularly during changes in walking direction, with noticeable peaks corresponding to turns or adjustments in heading.

Pitch and roll signals remain relatively stable, with minor fluctuations, reflecting slight postural adjustments and natural oscillations during walking.

The linear acceleration components (LIAx, LIAy, LIAz) show periodic patterns corresponding to the repetitive nature of gait cycles, with peaks and troughs indicating acceleration and deceleration phases during each step.

Together, these signals demonstrate the dynamic interaction between the user and the robotic walker during straight and curved walking paths, providing the basis for subsequent correlation and dimensionality reduction analyses.

Building upon the observed signal patterns, we further examined basic gait characteristics to estimate clinically relevant spatial parameters.

In addition to analyzing orientation and acceleration signals, we computed the average step length as a basic spatio–spatial gait parameter. The number of steps recorded during each trial was used along with the known walking path lengths—2.8 m for the straight path and 9.6 m for the rectangular path—to estimate the step length for each participant under both conditions.

The resulting average step lengths ranged from 0.29 m to 0.42 m. These values are consistent with typical ranges reported in the literature for older adults using assistive devices, where step lengths often fall between 0.30 m and 0.45 m. This analysis confirms that even with non-invasive sensors and simple processing, the system can extract clinically relevant gait parameters. Table 2 presents the results.

Due to the high dimensionality and multivariate nature of the collected sensor data (including orientation angles, linear accelerations, TOF distances, and wheel speeds), dimensionality reduction was applied to facilitate the analysis and visualization of user behavior patterns. Principal Component Analysis (PCA) was employed to identify dominant patterns and reduce redundancy by transforming correlated variables into uncorrelated principal components. Additionally, t-Distributed Stochastic Neighbor Embedding (t-SNE) was used to uncover local data structures and subtle groupings in the user interaction data that are not easily captured by linear methods. These techniques support the identification of user-specific motion signatures and contribute to understanding behavioral variability in different walking conditions. The dimensionality reduction results demonstrated that the system effectively distinguishes individual gait patterns and test conditions, confirming the analytical value of combining PCA and t-SNE in understanding multimodal sensor data.

**Figure 8:**
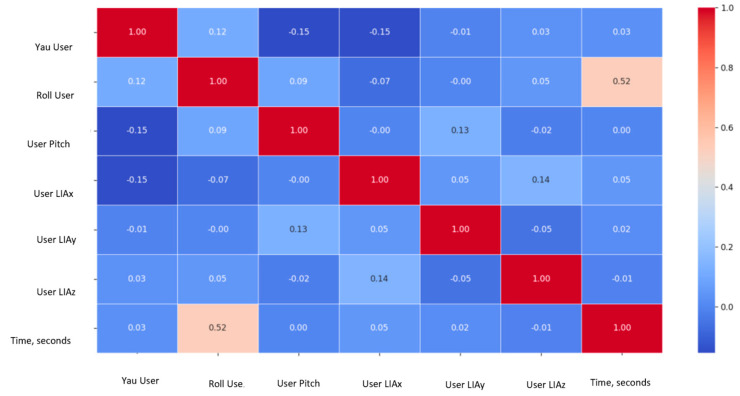
Correlation matrix among yaw, pitch, roll, LIAx, LIAy, LIAz, TOF distances, and wheel speed variables for the five test users.

Before applying PCA, a correlation matrix was generated to preliminarily explore the relationships between the collected sensor variables, including orientation angles, linear accelerations, distance measurements from TOF sensors, and wheel speed estimations. This correlation analysis allowed the identification of relationships between sensor modalities prior to dimensionality reduction, as shown in Figure 8.

**Figure 9:**
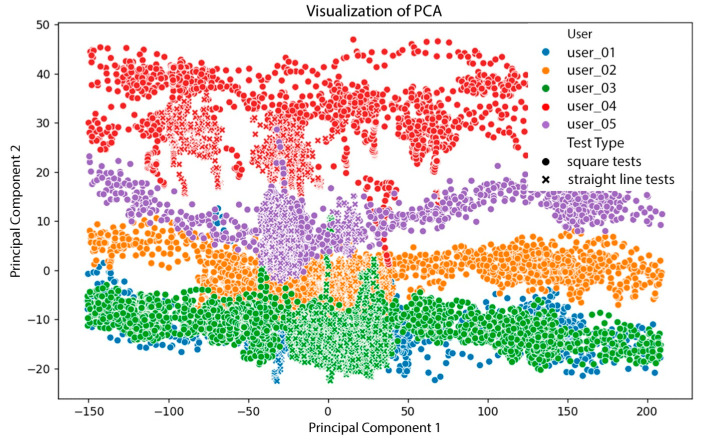
PCA Visualization of the five test users.

Subsequently, Principal Component Analysis (PCA) was applied to explore sensor data patterns and dimensional correlations. PCA is a dimensionality reduction technique that transforms a dataset of original variables into a smaller set of uncorrelated principal components that capture most of the data variance. In this context, PCA helps determine which combination of sensors and parameters best represents gait behavior, as seen in Figure 9.

The resulting PCA plots revealed the following insights:

1. Clear differentiation among users: Color-coded data points showed that each user had distinct interaction patterns with the walker, with identifiable regions in the PCA space.
2. Clustering by trial type: Markers (“×” for straight-line tests and circles for square-pattern tests) showed that test configuration influences the resulting sensor data distribution.
3. Non-uniform distribution across components: Some user groups were tightly clustered, while others were more dispersed, suggesting variability in motion consistency. For instance, User 4, with a known ligament injury, exhibited irregular gait patterns.
4. Sensor contribution: TOF and IMU sensors captured relevant and differentiable features, enabling the system to distinguish between individuals and movement conditions.

These results confirm that

1. The robotic walker reflects individual gait differences, as shown through PCA clustering.
2. Trial configuration (e.g., straight vs. square walking paths) affects sensor data distribution.

**Figure 10:**
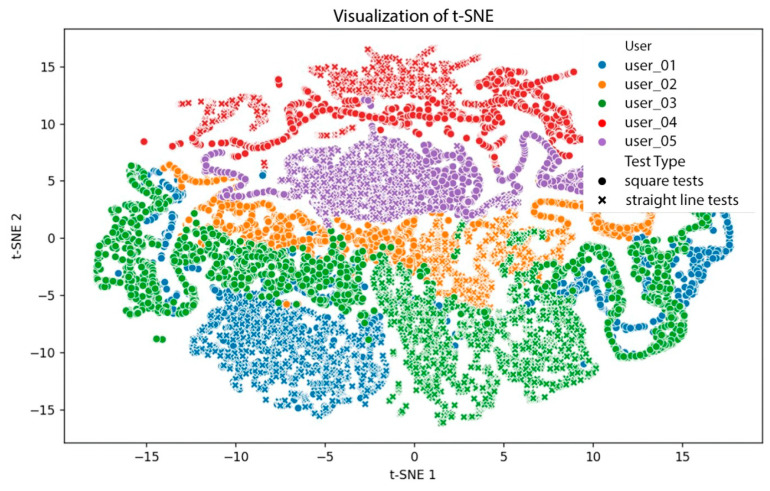
t-SNE Visualization.

To gain deeper insight, a t-Distributed Stochastic Neighbor Embedding (t-SNE) analysis was also performed; see Figure 10. As a non-linear dimensionality reduction technique, t-SNE is more effective than PCA at preserving local relationships and revealing natural groupings.

Key observations from the t-SNE visualization include the following.

1. User clustering: Five distinct user groups emerged, with more curved and compact structures than in PCA, confirming t-SNE’s ability to capture subtle differences.
2. Test type influence: Symbols representing straight and square walking tests revealed different distributions, with variation among users.
3. Transition continuity: Data exhibited smooth transitions between tests and users, although some sparse regions indicated lower variability.

These findings suggest that

1. t-SNE provides better visual discrimination of gait patterns than PCA.
2. Sensor data contains user-specific motion signatures.
3. Movement patterns differ by test type and user.

From the t-SNE visualization, it can be observed that

1. Users with high test variation: Users 01 and 03 showed significant distribution shifts between straight and square tests.
2. Users with low variation: Users 02 and 04 displayed consistent distributions, suggesting uniform gait across tests.
3. Test influence variability: Differences in how walking paths affect each user’s data indicate individual interaction styles with the walker.

**Figure 11:**
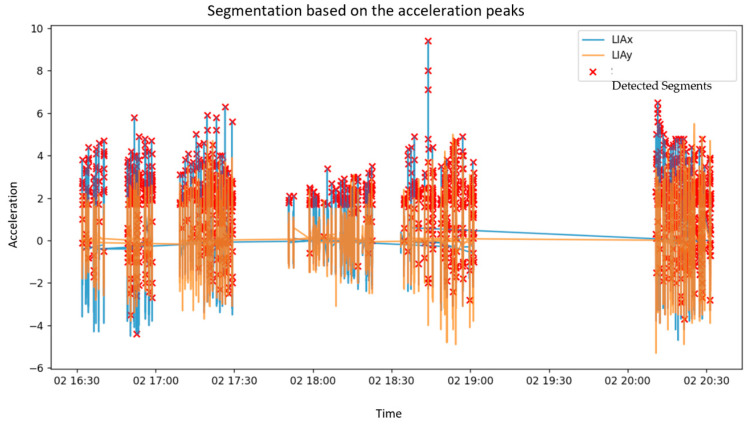
Segmentation Based on Acceleration Peaks.

Complementing the dimensionality reduction analysis, acceleration peaks were analyzed to segment gait cycles using LIAx and LIAy components, as seen in Figure 11. Red markers identified critical acceleration points.

**Figure 12:**
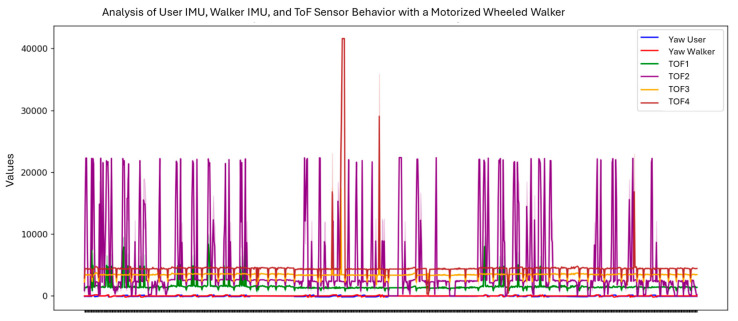
User and walker behavior without motor assistance.

The user’s and walker’s orientation evolution (yaw) was tracked along with TOF sensor readings, under both motorized and unmotorized test conditions. Yaw signals from the walker showed high variability, indicating multiple directional changes (see Figure 12). TOF sensors (TOF1–TOF4) reflected consistent oscillatory patterns with some peaks, marking acceleration bursts or sharp turns. This data revealed how the motorized system adapts to user movement and environmental interaction.

**Figure 13:**
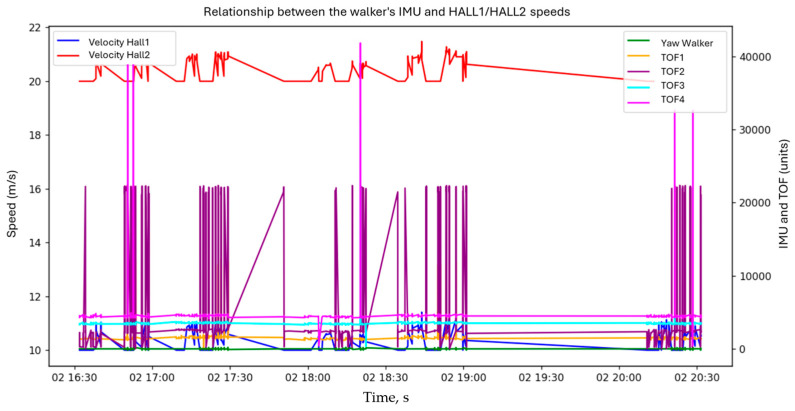
Relationship between walker IMU and wheel speeds (HALL1 and HALL2).

Figure 13 illustrates the correlation between walker orientation (yaw), wheel speeds (HALL1, HALL2), and TOF distances over time. Wheel speeds remained relatively stable with minor fluctuations, while TOF signals varied significantly, suggesting dynamic environmental responses. Low, stable yaw values indicated consistent directional heading.

Another plot compares the user and the walker’s yaw, pitch, and roll angles. Yaw signals exhibited notable spikes, likely due to sharp turns or wrapping artifacts. Roll and pitch signals remained relatively stable, with minor shifts possibly caused by posture adjustments.

The walker and user generally showed synchronized orientation behavior, confirming system responsiveness. However, in-depth comparisons revealed

1. Yaw differences: The walker showed greater angular variation.
2. Pitch trends: The user and walker shared similar inclination trends, with more fluctuation in the user.
3. Roll response: The user’s lateral movement exceeded the walker’s, reflecting natural postural compensation.

Finally, the relationship between walker orientation and TOF readings over time revealed sharp yaw changes (some due to incomplete wrapping correction), while TOF signals indicated distance variability during motion. This analysis confirmed the sensitivity of the sensing system and highlighted areas for refinement in orientation tracking and spatial awareness.

**Figure 14:**
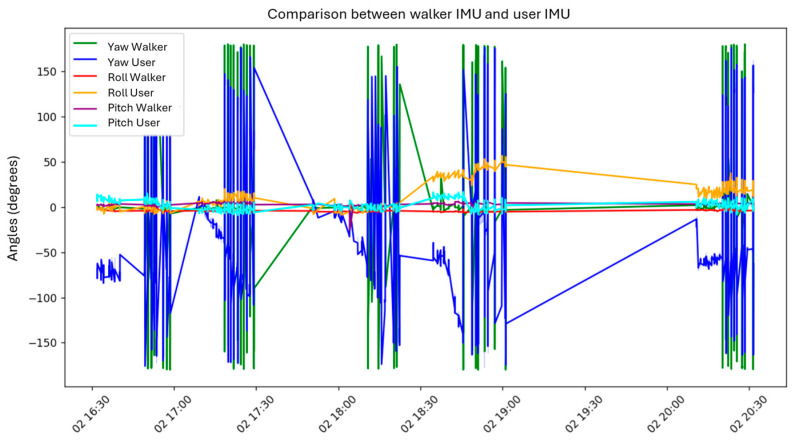
IMU comparison between the walker and the user.

Figure 14 shows that yaw signals from both the user and the walker (blue and green lines) exhibit significant fluctuations, including abrupt transitions likely caused by directional changes or wrapping effects in the orientation data. In contrast, the roll and pitch values for both systems (orange, red, and light blue) remain more stable, with minor oscillations. These variations may correspond to subtle postural adjustments by the walker or the user.

Figure 14 offers insight into the interaction dynamics between the user’s posture and the walker’s motion. By identifying moments of heightened orientation variability, the figure supports the evaluation of user–walker synchronization and highlights potential misalignments in gait behavior. In the case shown, the orientation trends of both the user and the walker appear nearly identical, indicating analogous behavior between the two systems.

These results confirm that the implemented robotic walker is capable of accurately capturing user gait dynamics, environmental interactions, and synchronization between the user and the device. Based on these findings, the following conclusions summarize the system’s performance and potential future improvements.

## 4. Conclusions

This study reports the experimental evaluation of a robotic walker prototype integrating multi-sensor systems (IMU, TOF, and pressure sensors) with motorized assistance, aimed at enhancing user mobility and safety. The complete integration of sensing, control, and motorization modules was validated through a series of real-world trials involving five test users with diverse physical profiles.

Principal Component Analysis (PCA) and t-Distributed Stochastic Neighbor Embedding (t-SNE) techniques were applied to explore the dimensionality and structure of the collected gait data. The results demonstrated the system’s capability to distinguish individual gait patterns and movement strategies, as well as to detect the influence of different walking configurations (straight-line and square-path tests). Both PCA and t-SNE confirmed that the combination of IMU and TOF data successfully captures relevant gait features, providing a rich set of indicators for analyzing user behavior.

The acceleration-based segmentation of gait cycles further enabled step detection and mobility parameter estimation without requiring invasive wearable systems. The walker’s onboard sensors reliably captured critical gait events, allowing for the calculation of parameters such as step length, step time, and cadence. Additionally, the dual IMU configuration (mounted on the user and walker) permitted the analysis of user–walker synchronization, revealing consistent orientation trends and postural adjustments during assisted walking.

Motorized support functions were evaluated through the monitoring of wheel speeds and walker orientation. Results showed stable control performance, with responsive adaptation to user movement patterns and obstacle proximity detected by TOF sensors. Minor yaw deviations and acceleration bursts were correctly reflected in the walker’s motion control, demonstrating the effectiveness of the integrated sensory feedback loop.

Overall, the experimental validation confirms that the developed robotic walker provides a safe, adaptive, and user-responsive platform for assisted mobility. The system’s modular design allows future enhancements, such as integrating real-time gait stability prediction algorithms or machine learning models for personalized assistance adjustment. Future work will also focus on expanding the range of tested user profiles (including elderly and mobility-impaired individuals) and conducting long-term validation in real-world environments.

## Figures and Tables

**Table 1:** Demographic characteristics of the five participants involved in the experimental trials, including gender, height, and weight.

Participant	Gender	Height (m)	Weight (kg)
User 1	Female	1.80	68
User 2	Male	1.83	98
User 3	Female	1.64	53
User 4	Male	1.77	83
User 5	Female	1.71	73

**Table 2:** Average number of steps and estimated step length per participant under straight and rectangular walking conditions. Step length was calculated using fixed path lengths of 2.8 m and 9.6 m, respectively.

Participant	Path Type	Avg. Steps	Avg. Step Length (m)
User 1	Straight	9.4	0.30
User 1	Rectangular	23.0	0.42
User 2	Straight	9.8	0.29
User 2	Rectangular	23.4	0.41
User 3	Straight	12.2	0.23
User 3	Rectangular	28.2	0.34
User 4	Straight	11.0	0.25
User 4	Rectangular	24.0	0.40
User 5	Straight	10.0	0.28
User 5	Rectangular	24.4	0.39

## Data Availability

Data sharing is not applicable to this article.
